# Technique for single-step lymphocyte isolation from an endoscopic biopsy specimen for the diagnosis of gastrointestinal lymphoma

**DOI:** 10.1016/j.mex.2020.101095

**Published:** 2020-10-10

**Authors:** Masaya Iwamuro, Takahide Takahashi, Natsuki Watanabe, Sizuma Omote, Katsunori Matsueda, Takehiro Tanaka, Daisuke Ennishi, Fumio Otsuka, Tadashi Yoshino, Hiroyuki Okada

**Affiliations:** a Department of Gastroenterology and Hepatology, Okayama University Graduate School of Medicine, Dentistry and Pharmaceutical Sciences, Okayama 700-8558, Japan; b Division of Medical Support, Okayama University Hospital, Okayama 700-8558, Japan; c Department of Pathology, Okayama University Graduate School of Medicine, Dentistry and Pharmaceutical Sciences, Okayama 700-8558, Japan; d Department of Gastrointestinal Oncology, Osaka International Cancer Institute, Osaka 541-8567, Japan; e Department of Hematology and Oncology, Okayama University Graduate School of Medicine, Dentistry and Pharmaceutical Sciences, Okayama 700-8558, Japan; f Department of General Medicine, Okayama University Graduate School of Medicine, Dentistry and Pharmaceutical Sciences, Okayama 700-8558, Japan

**Keywords:** Flow cytometry, Light chain restriction, Gastrointestinal lymphoma, Lymphocyte isolation

## Abstract

In this paper, we introduce a simplified, one-step procedure for lymphocyte isolation from an endoscopically biopsied fragment. For lymphocyte isolation, an endoscopically harvested specimen and 5 mL of normal saline solution were placed in a wire mesh strainer set in a porcelain bowl. To obtain the lymphocyte suspension, the solid specimen was crushed using the rubber portion of a plunger of a 10 mL injection syringe. Flow cytometry was performed using the lymphocyte suspension. For validating our methods, the one-step lymphocyte isolation technique was used to perform flow cytometry on samples from 23 patients with (n = 12) or without (n = 11) gastrointestinal lymphoma. Flow cytometry of light chain expression was performed in all patient samples (feasibility: 100%). Sensitivity was 83.3% (10/12) and specificity was 100% (11/11). In conclusion, lymphocytes isolated from a single endoscopic biopsy specimen using our simplified and quick procedure are suitable for flow cytometry. Considering that flow cytometry has an important advantage of providing the results on the examination day itself, the results of this study suggest that flow cytometric analysis using our single-step lymphocyte isolation technique can be potentially used to diagnose lymphoma in the gastrointestinal mucosa.

• We introduce a simplified, one-step procedure for lymphocyte isolation from an endoscopically biopsied fragment.
• Our technique is feasible for flow cytometric analysis in patients with gastrointestinal lymphoma as well as those with gastrointestinal lesions that are suspected to be lymphoma.

## Graphical Abstract

**Figure:**
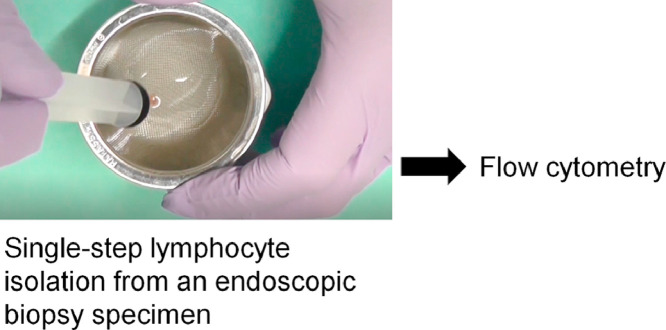

Specifications table

**Table:** 

Subject Area	Medicine and Dentistry
More specific subject area	*Gastrointestinal lymphoma, flow cytometry*
Method name	Single-step lymphocyte isolation from an endoscopic biopsy specimen
Name and reference of original method	*M. Iwamuro, K. Matsueda, T. Takahashi, S. Omote, T. Tanaka, D. Ennishi, F. Otsuka, T. Yoshino, H. Okada, An Endoscopic Biopsy Specimen Contains Adequate Lymphocytes for Flow Cytometric Analysis of Light Chain Expression in the Gastrointestinal Mucosa, Ann. Clin. Lab. Sci. 50 (2020) 348-353.*
Resource availability	*N/A*

## Method details

### Overview

Flow cytometric immunophenotyping is routinely used to aid the detection, classification, and monitoring of hematologic malignancies [1], [2], [3]. Because flow cytometry enables the rapid determination of surface antigens on the tested cells, the diagnosis of clonality and lymphoma subtypes can be completed on the day of examination [4,5]. Therefore, this method has an advantage over the conventional pathological evaluations using paraffin-embedded specimens, in that it can provide results faster than the conventional methods.

Recently, we reported that flow cytometric analysis of light chain expression in endoscopic biopsy specimens was feasible for the diagnosis of gastrointestinal B-cell lymphoma using two endoscopic biopsy fragments or even a single endoscopic biopsy fragment [6,7]. In our previous studies, we had used petri dishes, saline solution, a scalpel blade, tweezers, pipets, a pipet controller, a centrifugal separator, a tea strainer, and the plunger of a 10 mL syringe. Moreover, we performed the lymphocyte isolation procedure in a laminar flow cabinet. Because some of these apparatus are not available in most hospitals or clinics, we have modified the procedure so that it can be easily employed in a clinical setting. In this study, we introduce a simplified, one-step procedure for lymphocyte isolation from an endoscopically biopsied fragment. To the best of our knowledge, this is the simplest method reported thus far; lymphocyte isolation was completed within 2 min. In addition, laboratory wares and apparatus are not required for this procedure. Thus, lymphocyte isolation using our procedure can be performed in an endoscopy unit immediately after obtaining a biopsy specimen. This procedure would allow the widespread use of evaluating lymphocytes in the field of gastroenterology.

### One-step lymphocyte isolation

**Fig. 1:**
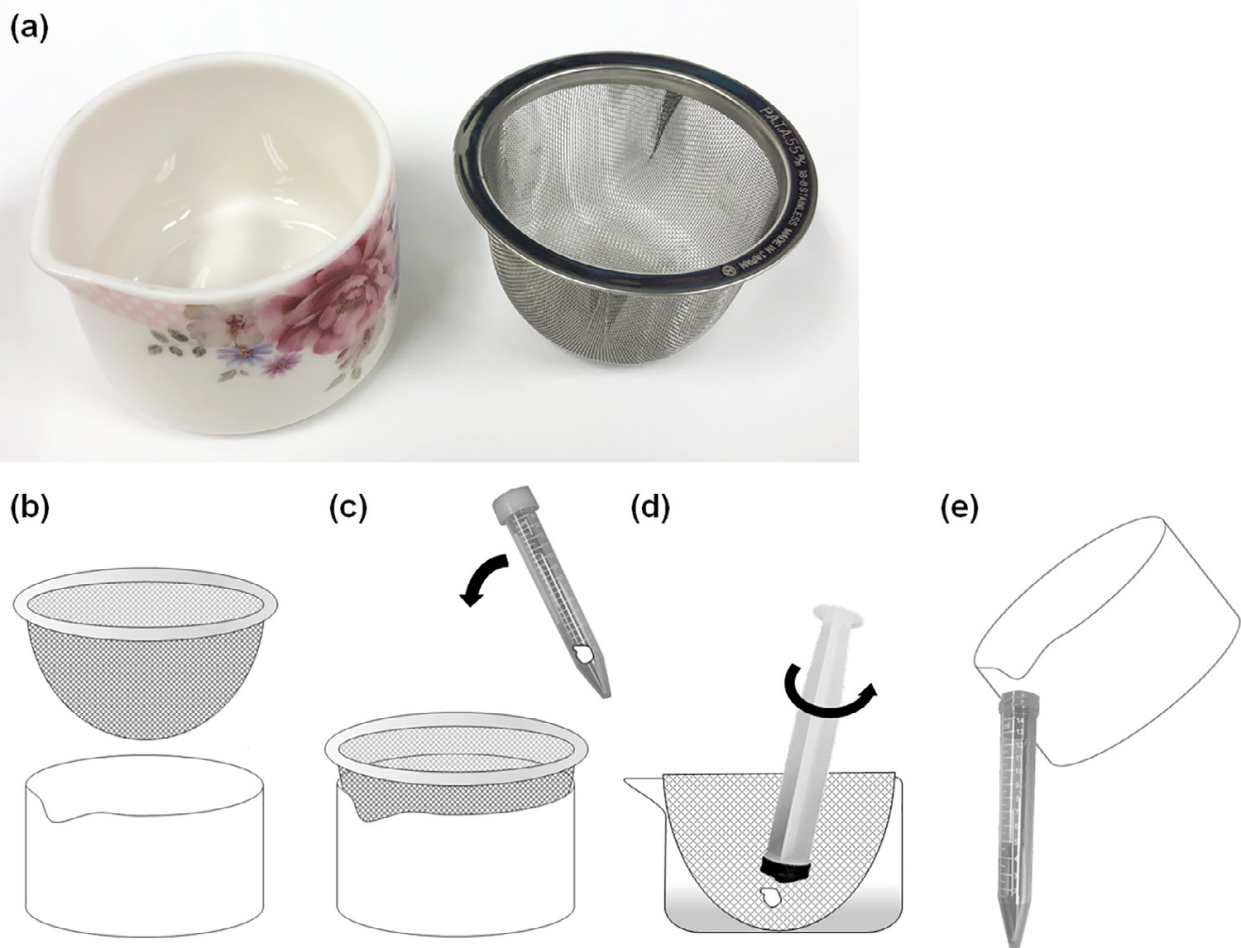
Scheme of the one-step lymphocyte isolation procedure. (a, b), Porcelain bowl with a spout and a wire mesh tea strainer were used; (c) One endoscopically harvested specimen was placed in a 15 mL conical centrifuge tube containing 5 mL of normal saline solution. A wire mesh strainer was set in a porcelain bowl, and saline solution containing specimen was decanted into it; (d) The tissue pieces were then crushed using the rubber portion of a plunger of a 10 mL injection syringe; (e) The saline solution with lymphocytes was then poured into the conical centrifuge tube.

For lymphocyte isolation, a porcelain bowl with a spout (Narumi Co. Aichi, Japan) and a wire mesh tea strainer (Sun Co., Niigata, Japan) were used (Fig. 1A, B). The diameter (55 mm) and height (40 mm) of the mesh strainer were equal to the internal diameter and depth of the porcelain bowl. The porcelain bowl and wire mesh strainer were sterilized by autoclaving prior to use. During endoscopy, a single specimen was obtained using disposable biopsy forceps (EndoJaw™, FB-230K, Olympus, Tokyo, Japan). First, one endoscopically harvested specimen was placed in a 15 mL conical centrifuge tube containing 5 mL of normal saline solution. Next, a wire mesh strainer was set in a porcelain bowl and saline solution containing the specimen was decanted into it (Fig. 1C). The conical centrifuge tube was mixed by inversion before decanting to ensure the removal of the specimen from the bottom of the tube. The solid specimens were then crushed using the rubber portion of a plunger of a 10 mL injection syringe (Fig. 1D). To obtain the lymphocyte suspension, the sample was soaked in saline solution during grinding. Fibrous residues remained on the wire mesh tea strainer. Subsequently, the wire mesh tea strainer was removed and discarded. The saline solution containing lymphocytes was then poured into a conical centrifuge tube (Fig. 1E).

### Flow cytometric analysis

Flow cytometry was performed according to the guidelines for analyzing surface antigens on hematopoietic malignant cells, issued by the Japanese Committee for Clinical Laboratory Standards (JCCLS) [8]. Immediately before flow cytometry, samples were filtered using a 100 μm pore mesh strainer (Partec CellTrics, Sysmex, Kobe, Japan) and centrifuged at 400 × g for 5 min at room temperature. The cells were washed with phosphate-buffered saline solution and centrifuged again. The supernatant was removed, and the pellet was mixed with monoclonal antibodies, diluted at the optimal concentration according to the manufacturer's instructions. The sample was kept in the dark for 15 min at room temperature. Monoclonal antibodies against CD45 (J33; Beckman Coulter, Pasadena, CA, USA), CD19 (J3-119; Beckman Coulter), CD20 (B-Ly1; Dako, Santa Clara, CA, USA), CD10 (ALB1; Beckman Coulter), CD3 (UCHT1; Beckman Coulter), and CD5 (BL1a; Beckman Coulter), were used and polyclonal antibodies were used for surface κ (Polyclonal F(ab’)2; Dako), and λ (Polyclonal F(ab’)2; Dako) chains. Next, erythrocytes were lysed by adding VersaLyse Lysing Solution (Beckman Coulter). After 10 min, the sample was diluted with phosphate-buffered saline solution and centrifuged at 400 × g for 5 min at room temperature. The pellet was resuspended in 500 μL phosphate-buffered saline solution and used for flow cytometry. The immunostained cells were analyzed by FACScan (Navios flow cytometer, Beckman Coulter) using Kaluza analysis software version 1.3 (Beckman Coulter).

CD45, originally known as leukocyte common antigen, is used as a marker of hematopoietic cells except erythrocytes and platelets. CD20 is acquired during the late stages of B-cell lymphogenesis, and its expression is lost upon plasma cell differentiation. On the other hand, the surface expression of CD19 is highly regulated throughout B-cell development and maturation until the loss of its expression during differentiation into plasma cells. Therefore, CD45 was used as a leukocyte lineage-specific antigen and CD19 and CD20 are used as B-cell lineage-specific antigens. Because B-cell lymphomas typically occur after transformation and subsequent clonal expansion of specific lymphocytes, neoplastic cells express only one class of immunoglobulin containing either κ or λ light chain. Therefore, light chain expression in a mature B-cell proliferation can be used as a surrogate marker to help diagnose B-cell lymphoma. Based on these principles, in this study, B-cell clonality was determined using the κ:λ ratio; the κ and λ light chain expression was quantified using a gated CD45 or CD20 population. Based on the previously published criteria for the flow cytometric analysis of restricted light chains, a κ:λ ratio within 0.5–3.0 was defined as negative for light chain restriction [6,^7^,[9], [10], [11].

The one-step lymphocyte isolation procedure is shown in Supplementary Video 1.

## Method validation

### Overview

**Fig. 2:**
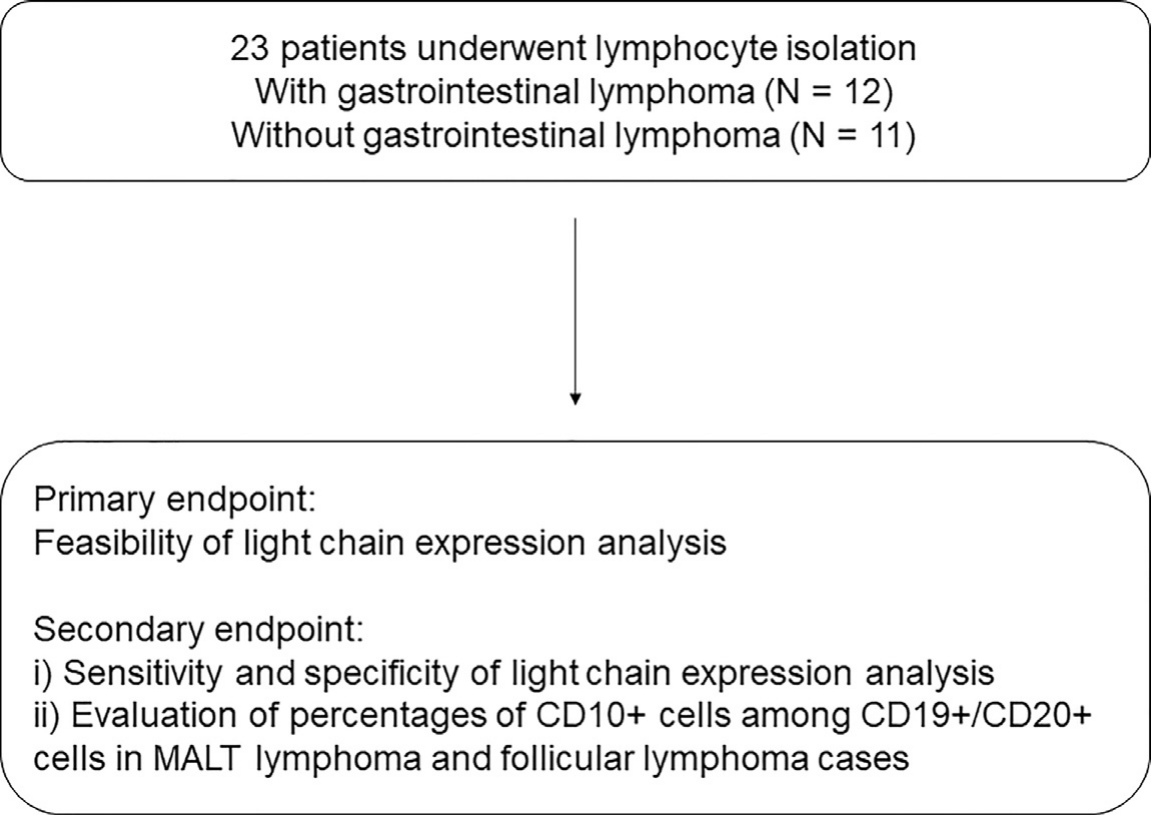
A flow diagram summarizing the study. MALT lymphoma, extranodal marginal zone lymphoma of mucosa-associated lymphoid tissue.

For the conventional pathological analysis, a specimen was retrieved from the target lesion using disposable biopsy forceps. Based on the results of pathological diagnosis, patients were divided into lymphoma and benign groups. We analyzed two factors, i.e., the feasibility of the flow cytometric analysis of light chain expression (Fig. 2), and the sensitivity and specificity of the flow cytometric analysis of light chain expression, which were determined by comparing the results with those of pathological diagnosis. In addition, the percentage of CD10+ cells among CD19+/CD20+ cells in the extranodal marginal zone of lymphoma in mucosa-associated lymphoid tissue (MALT lymphoma) patients—with light chain restriction—was compared with those of follicular lymphoma since CD10 is commonly expressed on the surface of follicular lymphoma cells, and MALT lymphoma cells are generally negative for CD10.

### Patients and ethics approval

Flow cytometry was performed using the one-step lymphocyte isolation procedure with endoscopic biopsy specimens obtained from 23 patients between April 2019 and March 2020 at Okayama University Hospital (Okayama, Japan). In this prospective study, patients with previously diagnosed gastrointestinal lymphoma or those with gastrointestinal lesions suspected to be lymphoma were included.

Patients were prospectively registered and analyzed for this study. Flow cytometric analyses were performed as part of the standard clinical practice. Therefore, the need for written informed consent was waived. This study was approved by the ethics committee of Okayama University Hospital and adhered to the principles of the Declaration of Helsinki. The study protocol was registered in the UMIN Clinical Trials Registry (UMIN000027730).

### Results

The characteristics of the patients (13 women and 10 men) are shown in Table 1. The median age was 65 years (range: 45–82 years). Biopsy sites included the stomach (n = 11), duodenum (n = 2), jejunum (n = 1), ileum (n = 2), cecum (n = 3), colon (n = 3), and rectum (n = 1). The final pathological diagnoses were lymphoma in 12 patients and benign lesions in 11 patients. The lymphoma subtype included gastric MALT lymphoma (n = 6), duodenal follicular lymphoma (grade 1; n = 2), cecal diffuse large B-cell lymphoma (DLBCL, n = 1), colonic DLBCL (n = 1), colonic MALT lymphoma (n = 1), and colonic follicular lymphoma (grade 1; n = 1). Benign lesions consisted of lymphoid hyperplasia (stomach, n = 1; rectum, n = 1), eosinophilic gastritis (n = 1), erosive gastritis (n = 1), gastric cancer (n = 1), non-specific ileitis (n = 1), ulcerative colitis (n = 1), colon cancer (n = 1), and the remission stage of lymphoma (ileal follicular lymphoma, n = 2; gastric MALT lymphoma, n = 1).

**Table 1:** Characteristics of the enrolled patients

	N
Sex	
Female	13
Male	10
Mean age (range, years)	65 (45–82)
Biopsy sites	
Stomach	11
Duodenum	2
Jejunum	1
Ileum	2
Cecum	3
Colon	3
Rectum	1
Pathological diagnosis	
MALT lymphoma	7
Follicular lymphoma	3
DLBCL	2
Benign lymphoid hyperplasia	2
Cancer	2
Eosinophilic gastritis	1
Erosive gastritis	1
Non-specific ileitis	1
Ulcerative colitis	1
Remission stage of lymphoma	3

MALT lymphoma, extranodal marginal zone lymphoma of mucosa-associated lymphoid tissue; DLBCL, diffuse large B-cell lymphoma

**Fig. 3:**
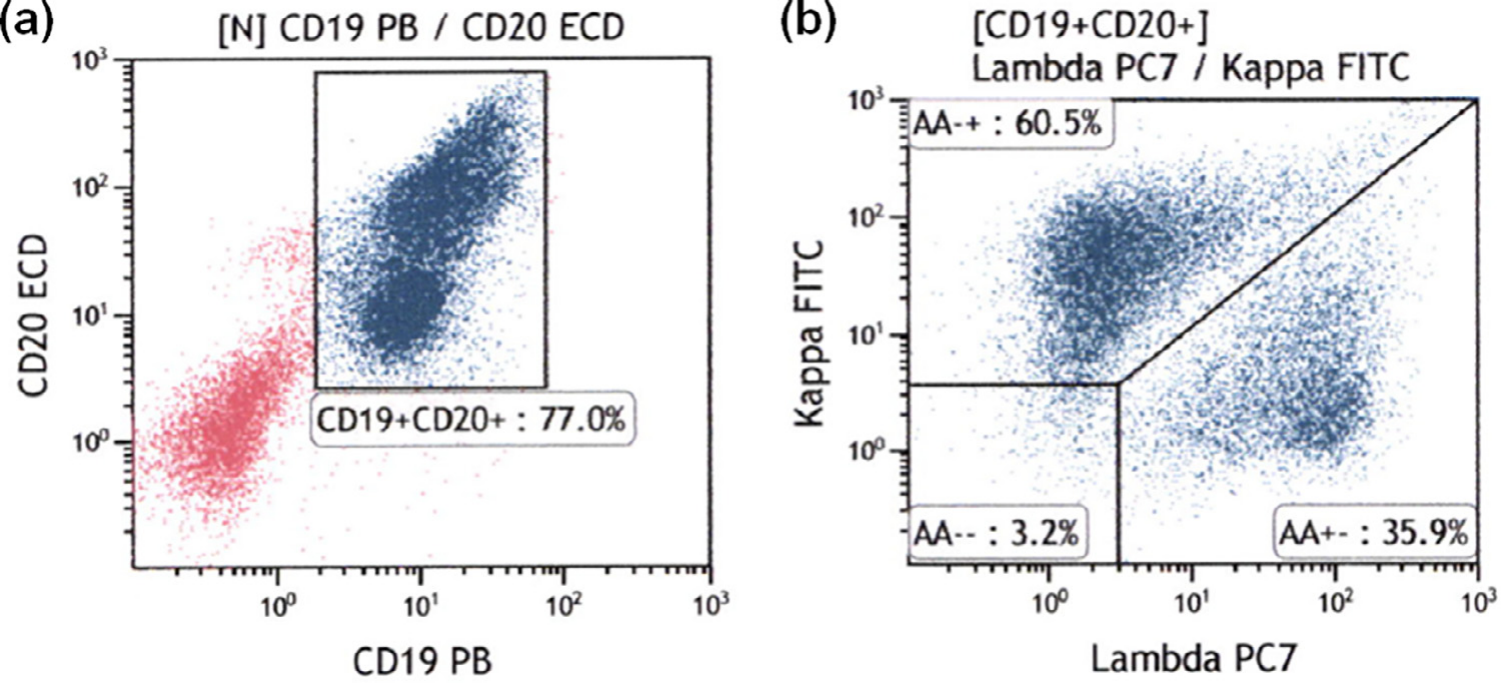
Flow cytometric results for rectal lymphoid hyperplasia. (a) CD19+/CD20+ cells were gated; (b) Light chain expression was analyzed and the κ/λ ratio was found to be 1.69 (normal range), indicating that the sample was negative for light chain restriction.

**Fig. 4:**
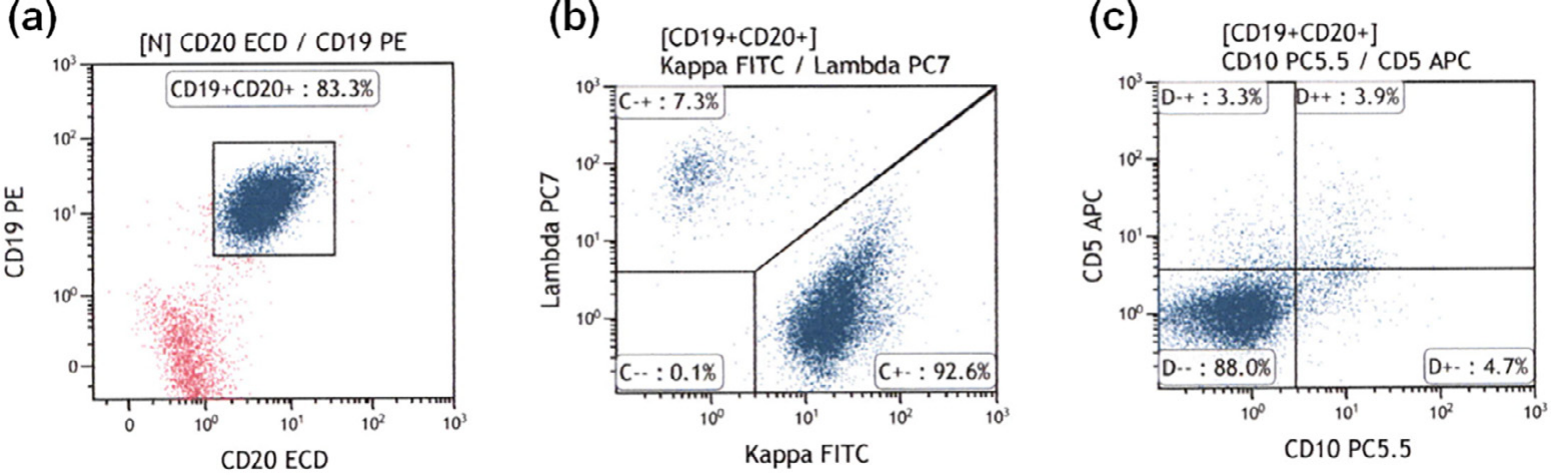
Representative flow cytometric results for gastric MALT lymphoma. (a) CD19+/CD20+ cells were gated; (b) There was predominant expression of the κ light chain, indicating that the sample was positive for light chain restriction; (c) The percentage of CD10+ cells was 7.4%.

**Fig. 5:**
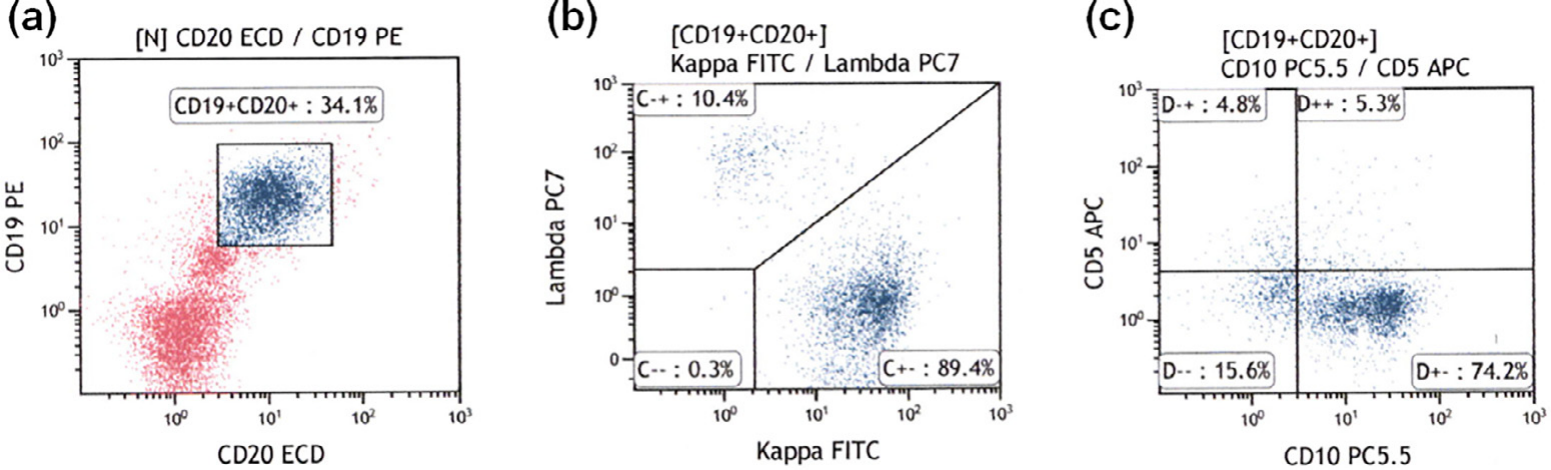
Representative flow cytometric results for duodenal follicular lymphoma. (a) CD19+/CD20+ cells were gated; (b) the κ/λ ratio was 8.58, indicating positive results for light chain restriction; (c) The percentage of CD10+ cells was 79.5%.

Flow cytometric analysis of light chain expression was performed in all patients. Therefore, the feasibility of flow cytometric analysis with the single-step lymphocyte isolation technique was 100%. The biopsy specimens of the 11 patients in the benign groups were light chain restriction negative (Fig. 3). The biopsy specimens from two patients with MALT lymphoma were light chain restriction negative, while those from the remaining 10 patients with lymphoma were light chain restriction positive (Figs. 4, 5). Overall, the sensitivity of light chain expression analysis for diagnosing lymphoma was 83.3% and specificity was 100% (Table 2).

**Table 2:** Cross-tabulation of the light chain expression analysis

	Lymphoma	Benign
Light chain restriction positive	10	0
Light chain restriction negative	2	11

In MALT lymphoma biopsy samples that were light chain restriction positive (n = 5), the percentages of CD10+ cells among CD19+/CD20+ cells were 4.0%, 8.6%, 8.7%, 9.2%, and 16.5% (Fig. 4C), and 79.5% and 96.3% in follicular lymphoma (n = 2) (Fig. 5C). Therefore, the follicular and MALT lymphoma cases could be correctly discriminated by flow cytometry, given that 20% is the threshold for CD10+ cells [10]. These results are in accordance with those of our previous study [5].

## Conclusion

In conclusion, we showed that lymphocytes isolated from a single endoscopic biopsy specimen using our simplified and quick procedure are suitable for flow cytometry. The sensitivity of the flow cytometric analysis of light chain expression was 83.3%, and specificity was 100%. Therefore, although flow cytometric analysis and pathological diagnosis are complementary methods for the diagnosis of gastrointestinal lymphoma, attainment of results on the day of examination is a strong advantage of flow cytometric analysis. We hope that this single-step lymphocyte isolation technique will assist in popularizing the use of flow cytometry among gastroenterologists and endoscopists for the diagnosis of gastrointestinal lymphoma.

## Declaration of Competing Interest

The authors declare that they have no known competing financial interests or personal relationships that could have appeared to influence the work reported in this paper.

## Appendix Supplementary materials

Image, video 1
